# Rise and fall of peroxisomes during Alzheimer´s disease: a pilot study in human brains

**DOI:** 10.1186/s40478-023-01567-0

**Published:** 2023-05-11

**Authors:** Eugen Semikasev, Barbara Ahlemeyer, Till Acker, Anne Schänzer, Eveline Baumgart-Vogt

**Affiliations:** 1 Division of Medical Cell Biology, Institute for Anatomy and Cell Biology, Justus-Liebig University, Aulweg 123, 35385 Giessen, Germany; 2 Institute of Neuropathology, Justus-Liebig University, Arndtstr. 16, 35392 Giessen, Germany; 3 Present Address: Department of Neurosurgery, University Hospital of Giessen, Klinikstr. 33, 35392 Giessen, Germany

**Keywords:** Catalase, Hippocampus, Neocortex, Neurodegenerative disorder, Peroxisome, PEX14, Pyramidal neurons

## Abstract

Peroxisomes are eukaryotic organelles that rapidly change in number depending on the metabolic requirement of distinct cell types and tissues. In the brain, these organelles are essential for neuronal migration and myelination during development and their dysfunction is associated with age-related neurodegenerative diseases. Except for one study analysing ABCD3-positive peroxisomes in neurons of the frontal neocortex of Alzheimer disease (AD) patients, no data on other brain regions or peroxisomal proteins are available. In the present morphometric study, we quantified peroxisomes labelled with PEX14, a metabolism-independent peroxisome marker, in 13 different brain areas of 8 patients *each either* with low, intermediate or high AD neuropathological changes compared to 10 control patients. Classification of patient samples was based on the official ABC score. During AD-stage progression, the peroxisome density decreased in the area entorhinalis, parietal/occipital neocortex and cerebellum, it increased and in later AD-stage patients decreased in the subiculum and hippocampal CA3 region, frontal neocortex and pontine gray and it remained unchanged in the gyrus dentatus, temporal neocortex, striatum and inferior olive. Moreover, we investigated the density of catalase-positive peroxisomes in a subset of patients (> 80 years), focussing on regions with significant alterations of PEX14-positive peroxisomes. In hippocampal neurons, only one third of all peroxisomes contained detectable levels of catalase exhibiting constant density at all AD stages. Whereas the density of all peroxisomes in neocortical neurons was only half of the one of the hippocampus, two thirds of them were catalase-positive exhibiting increased levels at higher ABC scores. In conclusion, we observed spatiotemporal differences in the response of peroxisomes to different stages of AD-associated pathologies.

**Graphical Abstract:**

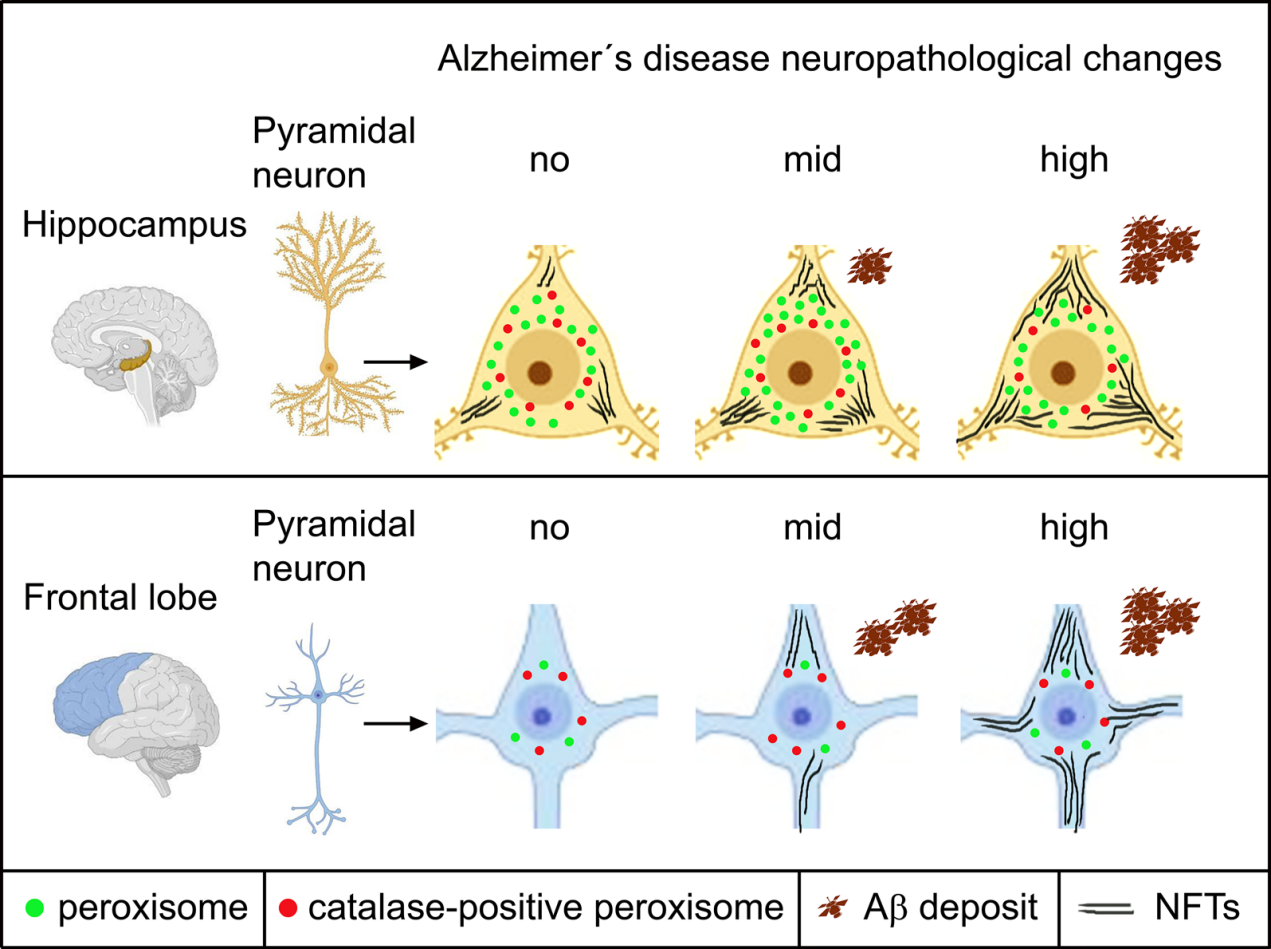

**Supplementary Information:**

The online version contains supplementary material available at 10.1186/s40478-023-01567-0.

## Introduction

AD is one of the most common neurodegenerative diseases, the rates of which is increasing with age with 5% at 75–79 years, 10% at 80–84 years and 25% at 90–94 years of age [63]. The disease AD subdivides according to the cause into the rare familial forms (5% of all cases with mutations in several genes, e.g. the amyloid precursor protein and secretase genes) and the more frequent sporadic idiopathic form (95% of all cases). Multiple factors such as the presence of the APOE4 allele, hypertension, diabetes type 2 and hypercholesterolemia increase the risk to develop AD [21]. Postmortem diagnosis and classification of human brain autopsy material [56] are based on the level of extracellular amyloid-β (Aβ; A score, Thal phases) [85], intracellular neurofibrillary tangles (NFTs, B score, Braak stages) [11] and neuritic plaques (C score, CERAD) [55]. Aβ plaques appear first in the frontal, occipital, and temporal neocortex (phase 1), thereafter in the area entorhinalis and hippocampus (phase 2), thalamus, striatum, inferior olive (phase 3), substantia nigra, medulla oblongata (phase 4) and finally in the pontine gray and cerebellum (phase 5) as shown by [85]. NFTs are present already decades before clinical symptoms are observed, starting in subcortical areas such as the amygdala, thalamus and hypothalamus (stages 0-I) [81], followed by the transentorhinal area (stage I), area entorhinalis and hippocampus (stage II), frontal, temporal (stages III-IV) and parietal and occipital neocortex (stage V) and striatum (stage VI) as analysed by [11]. According to the histopathologic assessment, the disease is classified into the ABC score indicating no, low, intermediate (mid) and high AD neuropathological changes (ADNC) [56]. The roles of Aβ and NFTs for AD etiopathogenesis are still under debate. It is assumed that an increase of the Aβ level over a certain threshold induces a profound deposition of NFTs in association with an activation of microglia cells and release of pro-inflammatory cytokines [28, 62, 86]. Vice-versa, PHF tau is supposed to mediate dendritic toxicity of Aβ oligomers [42] and to induce Aβ secretion in a kind of a vicious cycle [87]. The clinical status, however, correlates better with the distribution of NFT lesions than with Aβ deposits [19].

Peroxisomes are highly dynamic and ubiquitous organelles. Their number and size are tightly controlled by peroxisomal biogenesis proteins, named peroxins (or PEX proteins), regulating the de novo biogenesis and proliferation of pre-existing organelles, their functional maturation as well as degradation. These organelles play an important role in lipid homeostasis and are thus essential for a proper function of the lipid-rich brain [93]. For example, very-long chain fatty acids (VLCFAs), bioactive and pro-inflammatory lipid derivatives are transported into the peroxisomal matrix by ABC lipid transporters (subfamily D, e.g. ABCD3, formely called PMP70) and are there degraded by oxidation. Moreover, peroxisomes are involved in the synthesis of docosahexanoic acid (DHA) and precursors of cholesterol and ether lipids (e.g. plasmalogens) which regulate cell membrane fluidity, membrane protein signaling, myelination and formation of transmitter vesicles [44]. The peroxisomal matrix enzyme catalase together with reactive oxygen species (ROS)-trapping plasmalogens are strong defenses against oxidative stress [12]. Accordingly, the most severe form of hereditary peroxisome biogenesis disorders is the cerebro-hepato-renal (Zellweger) syndrome [92]. Patients with this devastating disease exhibit an impaired neuronal development such as migration defects, they suffer from epileptic seizures, general hypotonia and they die within the first year of life [18]. During aging and in neurodegenerative diseases, the function of peroxisomes has been suggested to be dysregulated as evidenced by changes in their abundance, defects of the import machinery or reduced levels of peroxisomal membrane proteins (PMPs) [44, 88]. In vitro experiments showed an early upregulation followed by a dramatic decrease of the lipid transporter ABCD3 and a continuous decrease of catalase during chronic exposure of primary rat cortical neurons to Aβ [16]. In a transgenic AD mouse model, the peroxisome density (as measured by the level of PEX14) decreased, whereas the levels of ABCD3, catalase and acyl-CoA oxidase 1 increased during the first 3 months with a return to control level at 6 to 18 months of age [24]. In postmortem human brains, namely in the frontal cortex of stage V-VI patients, Kou and colleagues [48] measured an increase in the density of ABCD3-positive peroxisomes in the soma of neurons, however a reduced number in the processes suggesting an impaired organelle transport. Biochemical analyses of the brain of AD in comparison to control patients further points to a disturbed peroxisomal lipid metabolism with increased levels of VLCFAs (> C22:0) [48] and reduced levels of DHA [4, 30, 53, 79] and plasmalogens [31].

In our broad morphometric study, we analyzed the numerical abundance of peroxisomes in brain autopsy samples of patients with no, low, mid and high ADNC. We quantified peroxisomal density in 13 different brain regions known also for their high peroxisome metabolic protein content [3, 34, 57, 58, 61, 73, 101] by immunofluorescence staining of the peroxisomal biogenesis protein PEX14, an optimal marker of this organelle for morphometric studies [34]. In addition, we comparatively analysed the density of catalase-positive peroxisomes in a subset of patients, mainly focussing on the 4 brain regions with most significant alterations of PEX14-labelled peroxisomes.

## Material and methods

### Postmortem human brain material

Tissue blocks of 8 human brains each either with low, mid or high ADNCs, 6 brains with tauopathy and 10 aged- and gender-matched controls were taken from the donation bank of the department of neuropathology. We used formalin-fixed brain samples with a postmortem interval of 1 to 5 days. The following regions were cut out of the brains and were later embedded in paraffin in a defined stereological pattern: the hippocampus, frontal, temporal, parietal and occipital neocortices, striatum, midbrain including the substantia nigra, pons, medulla oblongata including the inferior olive, and cerebellum (for details see Additional file 1: Fig. S1). Data of each patient including age, gender, ABC score (evaluated by two investigators) as well as clinical data are shown in the Additional files 6, 7: Tables S1 and S2, respectively. All patients signed a written informed consent and agreement that their brains—after death—will enter the brain donation bank of the Institute of Neuropathology to be used for diagnosis, research and teaching. All experiments have been approved by the ethic committee of the Justus Liebig University (AZ 07/09).

### Histopathological evaluation of formalin-fixed paraffin-embedded (FFPE) human brain tissue for the AD classification

**Fig. 1:**
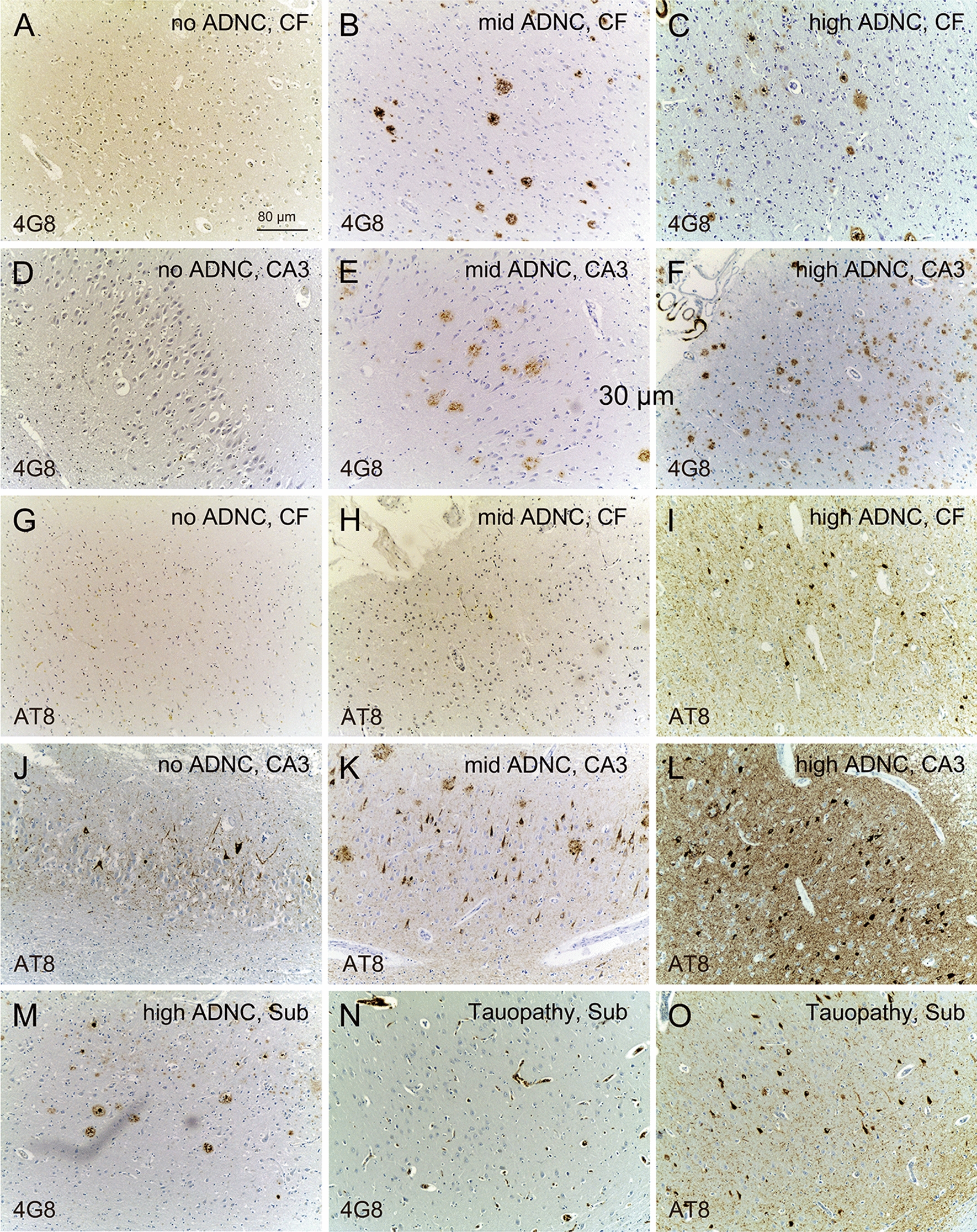
Immunohistochemical detection of Aβ including neuritic plaques and NFTs to classify the human brain samples to patients with no, low, mid and high ADNC. Light microscopy images of Aβ deposits (4G8, **a-f**), NFTs (AT8, **g-l**) and neuritic plaques (4G8-positive plaques with a dense core accompanied by AT8-positive abnormal neurites) in the frontal neocortex (CF), and hippocampal CA3 region (CA3). Whereas high amounts of Aβ (**m**) and NFTs (not shown) were found in the subiculum of patients with high ADNC, a high density of NTFs (**n**), in the absence of Aβ (**o**) was found in the subiculum (Sub) of the patient with tauopathy

Sections of 3–4 µm thickness from FFPE material of the frontal neocortex and hippocampus were taken to perform routine hematoxylin–eosin stain and detection of Aβ plaques using 0.1% thioflavin S (only in AD cases). In addition, immunohistochemistry was done in the fully automated BenchMark Ultra IHC slide staining system (Roche Diagnostics) using primary mouse antibodies against Aβ (1:10.000, 4G8, Covance, SIG-39220) or hyperphosphorylated tau (1:2000, AT8, Invitrogen, MN1026) [6] combined with ultraView Universal DAB detection kit (Roche Diagnostics, 760–500) and bluing reagent (Roche Diagnostics, 760–2037). Neuropathology of each patient was diagnosed in the years between 2009 and 2016 by the abundance of Aβ plaques and NFTs detected under a light microscope and thus before we started our study by 2 neuropathologists (not aware of the recent study design) and additionally controlled for correctness of the documented ABC score by the authors. The samples were classified as follows: patients with no (A0/B0-1; Fig. 1a, d, g, j), low (A1/B0-1 A2/B1; not shown), mid (A1-3/B2; Fig. 1b, e, h, k) and high (A2-3/B3; Fig. 1c, f, i, l, m) ADNC as well as patients with primary tauopathy (A0/B2; Fig. 1n, o). Details about each patient, e.g. sex, age, ABC score and abundances of Aβ plaques and NFTs in the frontal neocortex and hippocampus are given in Additional file 6: Table S1.

### Immunofluorescence staining to characterize the density and anti-oxidative function of peroxisomes in human brain tissue

Two-µm sections of FFPE brain samples containing the hippocampus, 4 different neocortical areas, striatum, substantia nigra, pons, inferior olive and cerebellum were cut and only every third section was taken for the double immunofluorescence stainings for the 4 combinations of PEX14 and catalase each either with Aβ or PHF tau. Prior to the staining, paraffin was removed by incubating the slides in xylene followed by rehydration through a descending graded alcohol series. Antigen retrieval was performed by microwave irradiation (3 × 5 min) at 900 Watt in 10 mM citrate buffer (pH 6.0) and subsequent cooling to room temperature for 30 min. Non-specific binding sites were blocked with 4% bovine serum albumin (BSA) in phosphate-buffered saline (PBS) with 0.05% Tween 20 for 2 h. Thereafter, the sections were incubated with the primary antibodies diluted in 1% BSA in PBS with 0.05% Tween 20 overnight at room temperature. The following primary antibodies were used: 1:5000 PEX14 (gift from Denis Crane, Griffith University, made in rabbit), 1:300 catalase (Proteintech, 21,260–1-AP, made in rabbit), 1:10,000 Aβ, (4G8, Covance, SIG-39220, made in mouse) and 1:2000 PHF (AT8, Invitrogen, MN1026, made in mouse). The next day, sections were washed with PBS and donkey anti-rabbit IgGs coupled with Alexa488 (1:300, Invitrogen, A1055) or anti-mouse IgGs coupled with TexasRed (1:300, Vector Laboratories, TI-2000) were added for 2 h. Thereafter, sections were again washed and nuclei were counterstained with DAPI or TOPRO-3 iodide in a 1:750 dilution in PBS from a stock of 1 mg/ml. Slides were mounted with a mixture (3:1) of Mowiol® 4–88 mounting medium and n-propylgallate as fading agent.

### Analysis of the intraneuronal density of peroxisomes

**Fig. 2:**
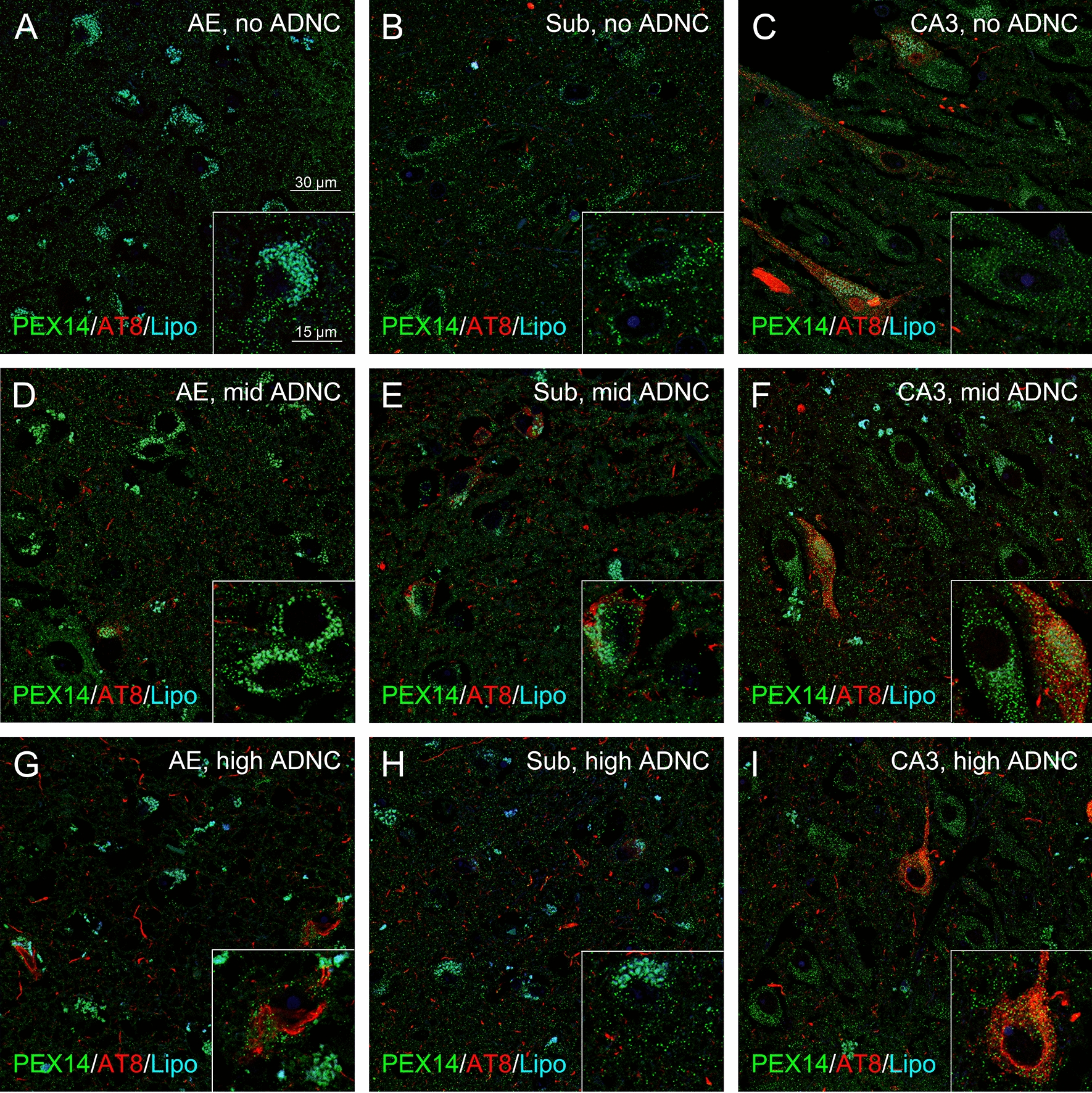
Peroxisome density of pyramidal neurons in the subiculum and CA3 region was higher in patients with mid in comparison to those with no and high ADNC. Representative photomicrographs of immunostainings for the peroxisomal marker PEX14 (green), hyperphosporylated tau (AT8, red) together with autofluorescent lipofuscine granules (turquoise) in neurons of the area entorhinalis (AE), subiculum (Sub) and hippocampal CA3 region (CA3)

**Fig. 3:**
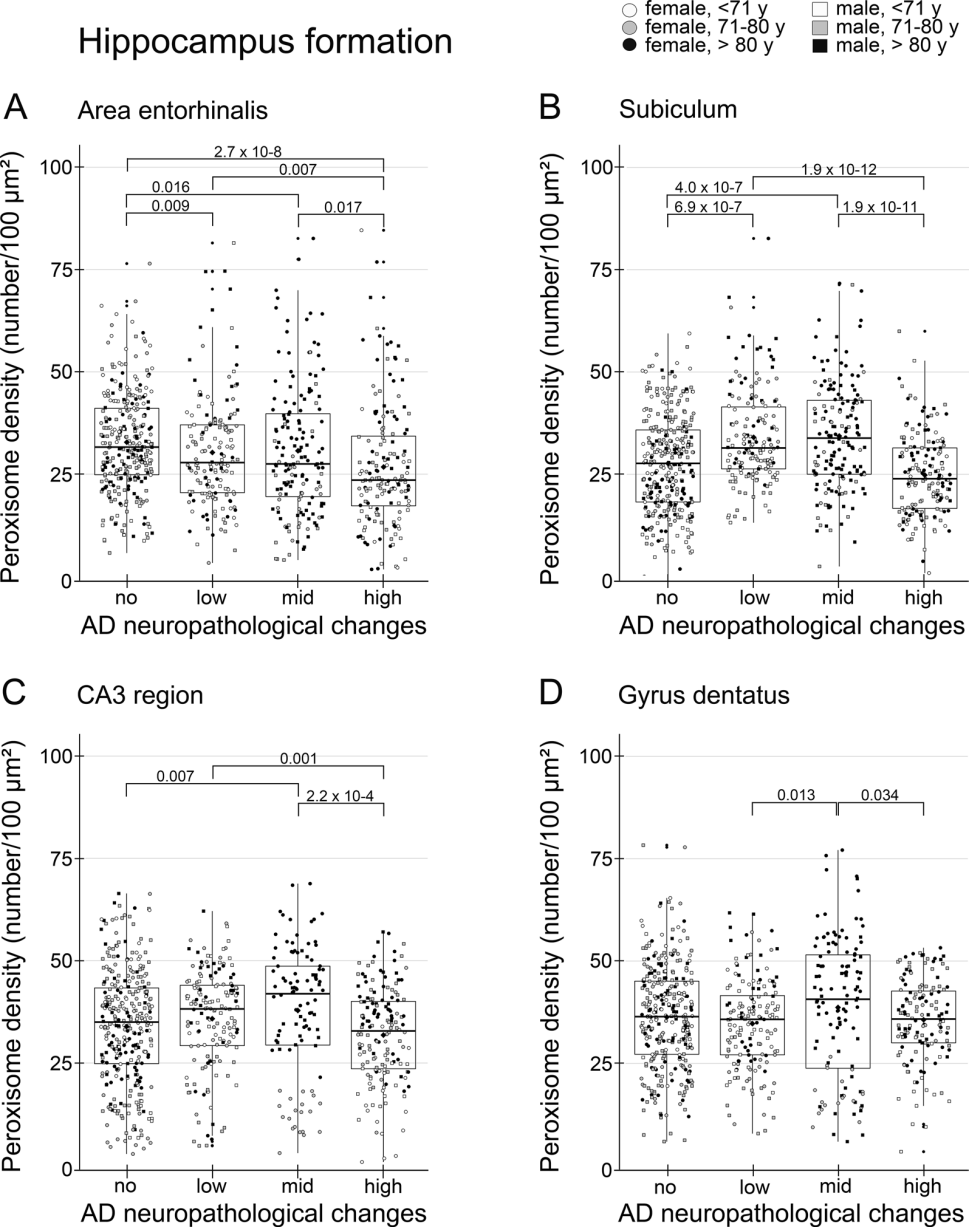
Initial rise and fall of peroxisome density in the subiculum and CA3 region of the hippocampus at ongoing stages of AD. Data were obtained using PEX14 immunofluorescence images running through a self-written ImageJ macro-tool for counting particles and measuring the cytosolic area. Ten individual neurons were analysed from each patient and plotted as a point on the graph. Crossbar = median value, black diamond in the box center = mean value, vertical lines above and below each box = SD values

**Fig. 4:**
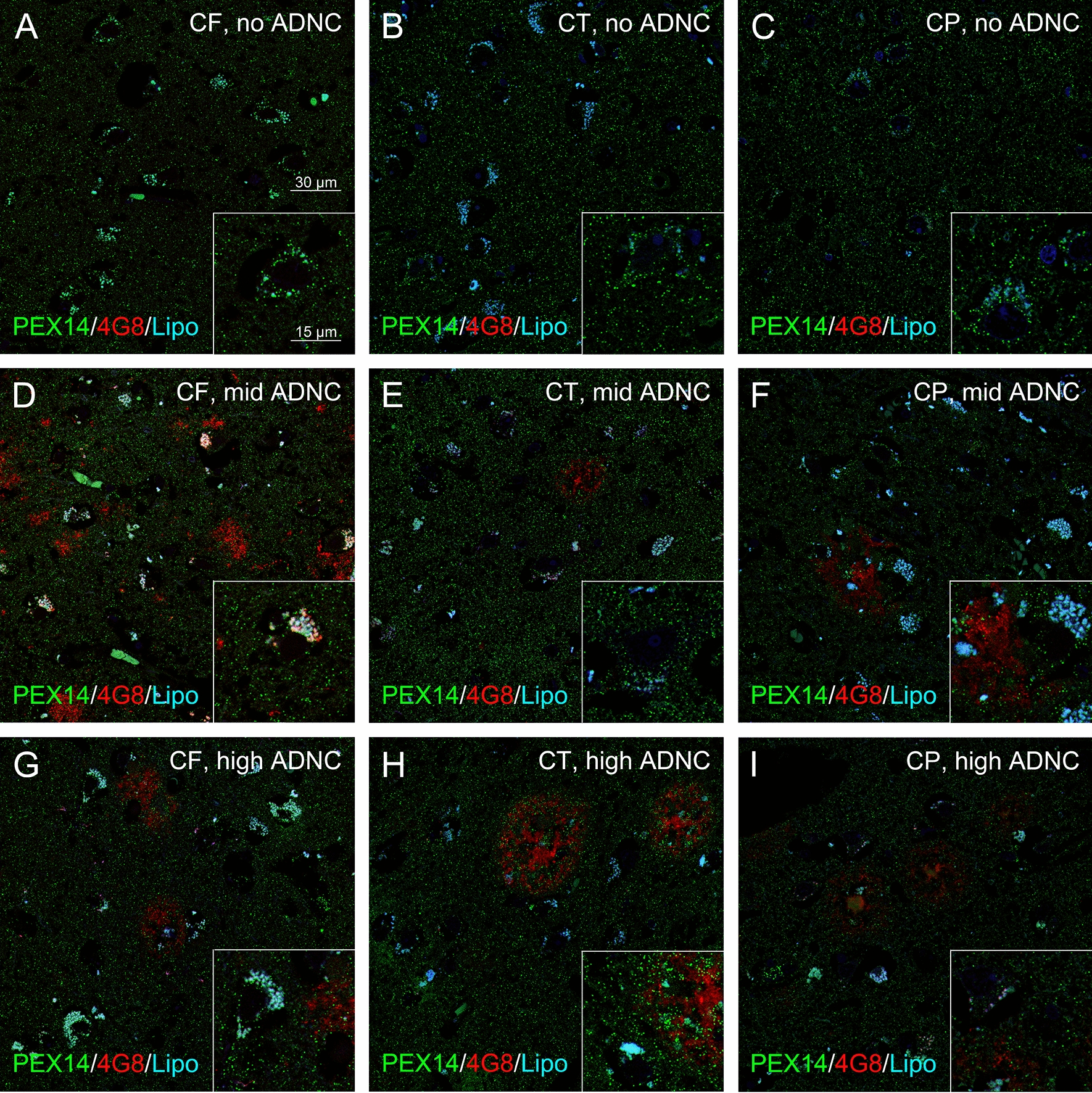
Minor decrease in the peroxisome density of pyramidal neurons in the neocortex of patients with high ADNC. Representative photomicrographs of immunostainings for the peroxisomal marker PEX14 (green), Aβ plaques (4G8, red) together with autofluorescent lipofuscine granules (turquoise) in neurons of the frontal (CF), temporal (CT) and parietal (CP) cortices

**Fig. 5:**
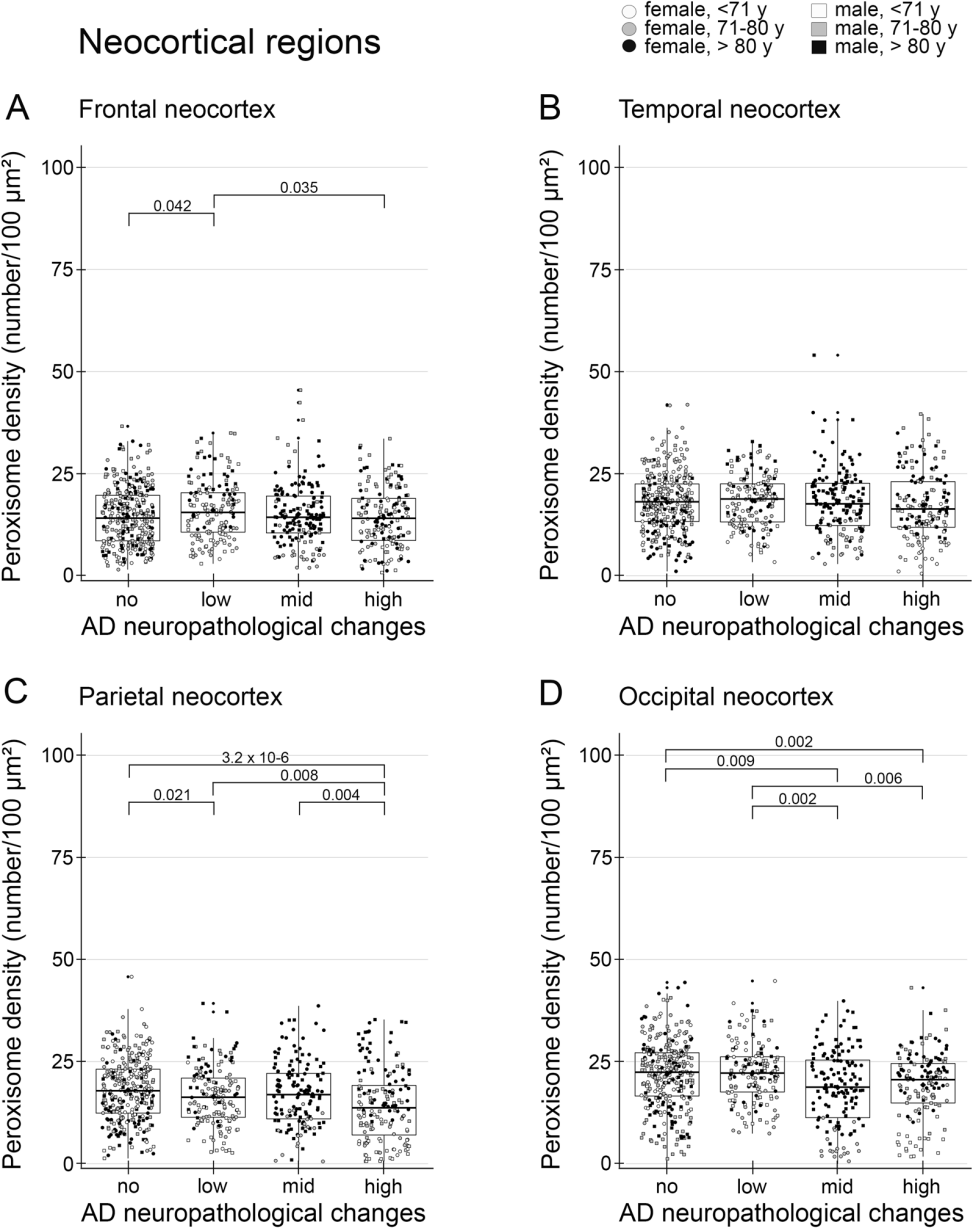
The peroxisome density decreased in pyramidal neurons of the frontal, temporal, parietal and occipital lobe. Data were obtained using PEX14 immunofluorescence images running through a self-written ImageJ macro-tool for counting particles and measuring the cytosolic area. Ten individual neurons were analysed from each patient and plotted as a point on the graph. Crossbar = median value, black diamond in the box center = mean value, vertical lines above and below each box = SD values

**Fig. 6:**
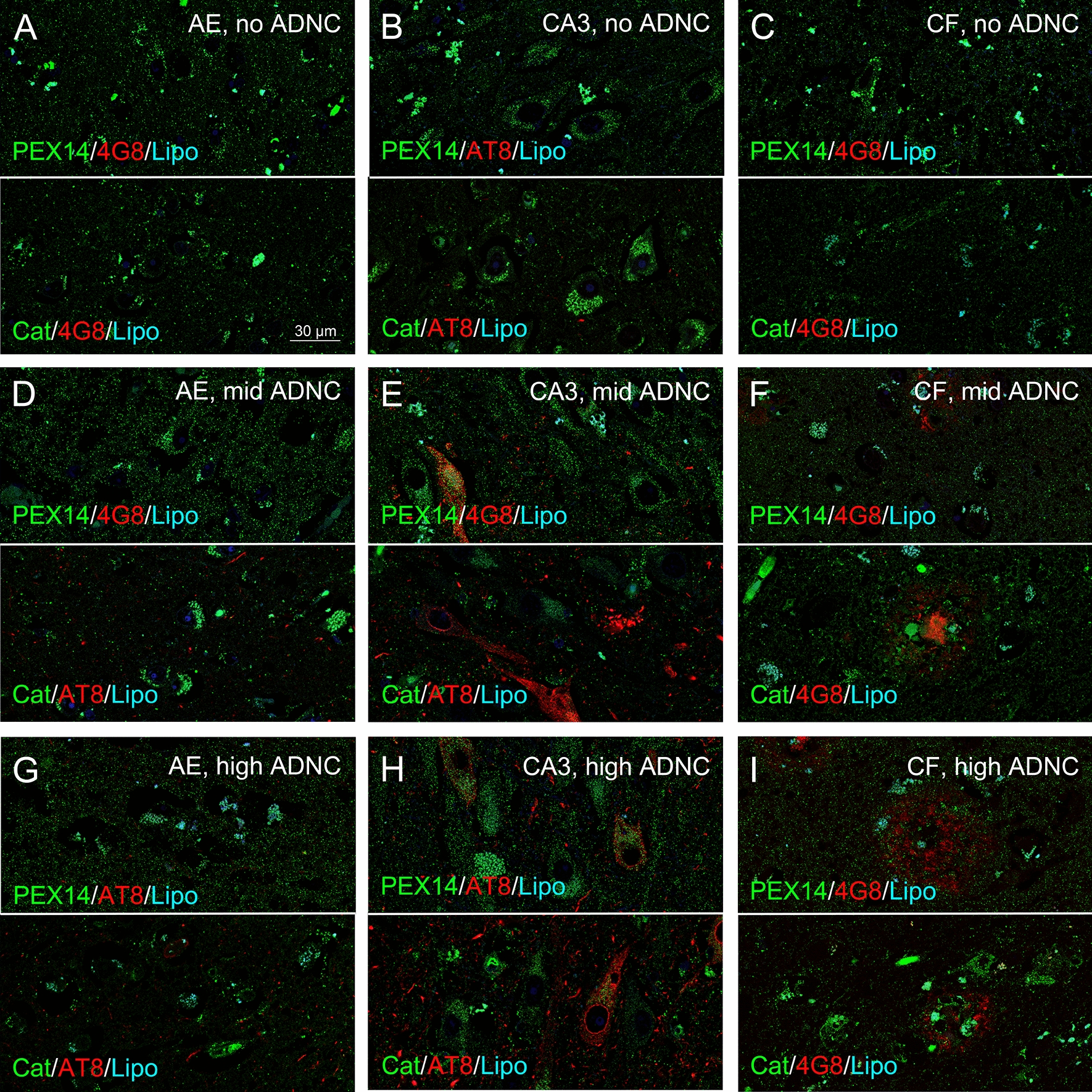
A concomitant increase in PEX14- and catalase-positive peroxisome densities during AD-stage progression occurred only in the frontal neocortex, but not in the hippocampus. Representative photomicrographs of immunostainings for the peroxisomal marker PEX14 (PEX14, green) or catalase (Cat, green), Aβ plaques (4G8, red) and hyperphosphorylated tau (AT8, red) together with autofluorescent lipofuscine granules (turquoise) in neurons of area entorhinalis (AE), hippocampal CA3 region (CA3) and the frontal neocortex (CF)

**Fig. 7:**
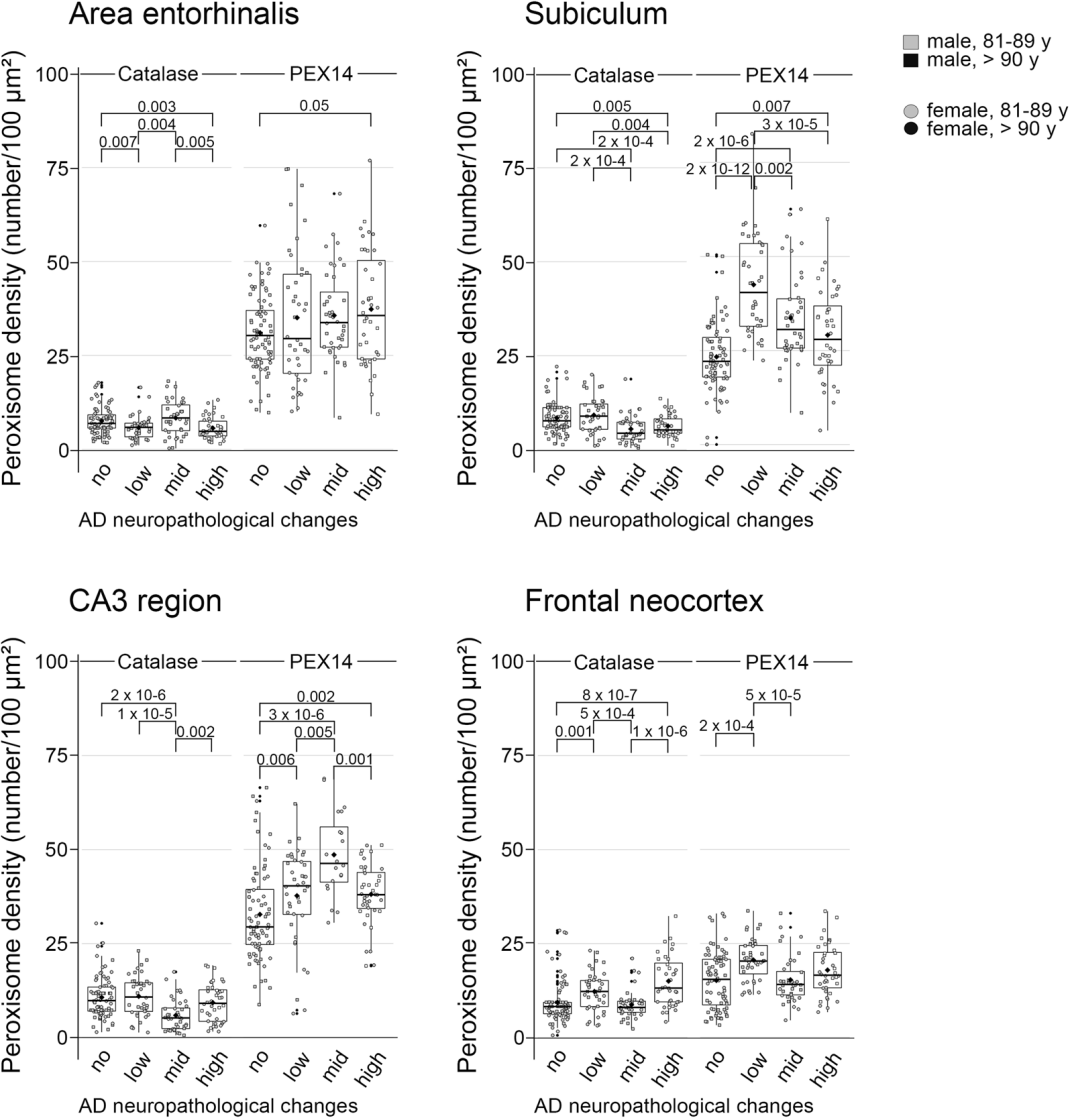
In a subset of patients (> 80 years of age), the peroxisome density increased during AD-stage progression in all 4 regions analyzed, a concomitant increase in the density of catalase-positive peroxisomes was detected only in the frontal neocortex, but not in the hippocampus. Data were obtained using PEX14 or catalase immunofluorescence images running through a self-written ImageJ macro-tool for counting particles and measuring the cytosolic area. Ten individual neurons were analysed from each patient and plotted as a point on the graph. Crossbar = median value, black diamond in the box center = mean value, vertical lines above and below each box = SD values

**Fig. 8:**
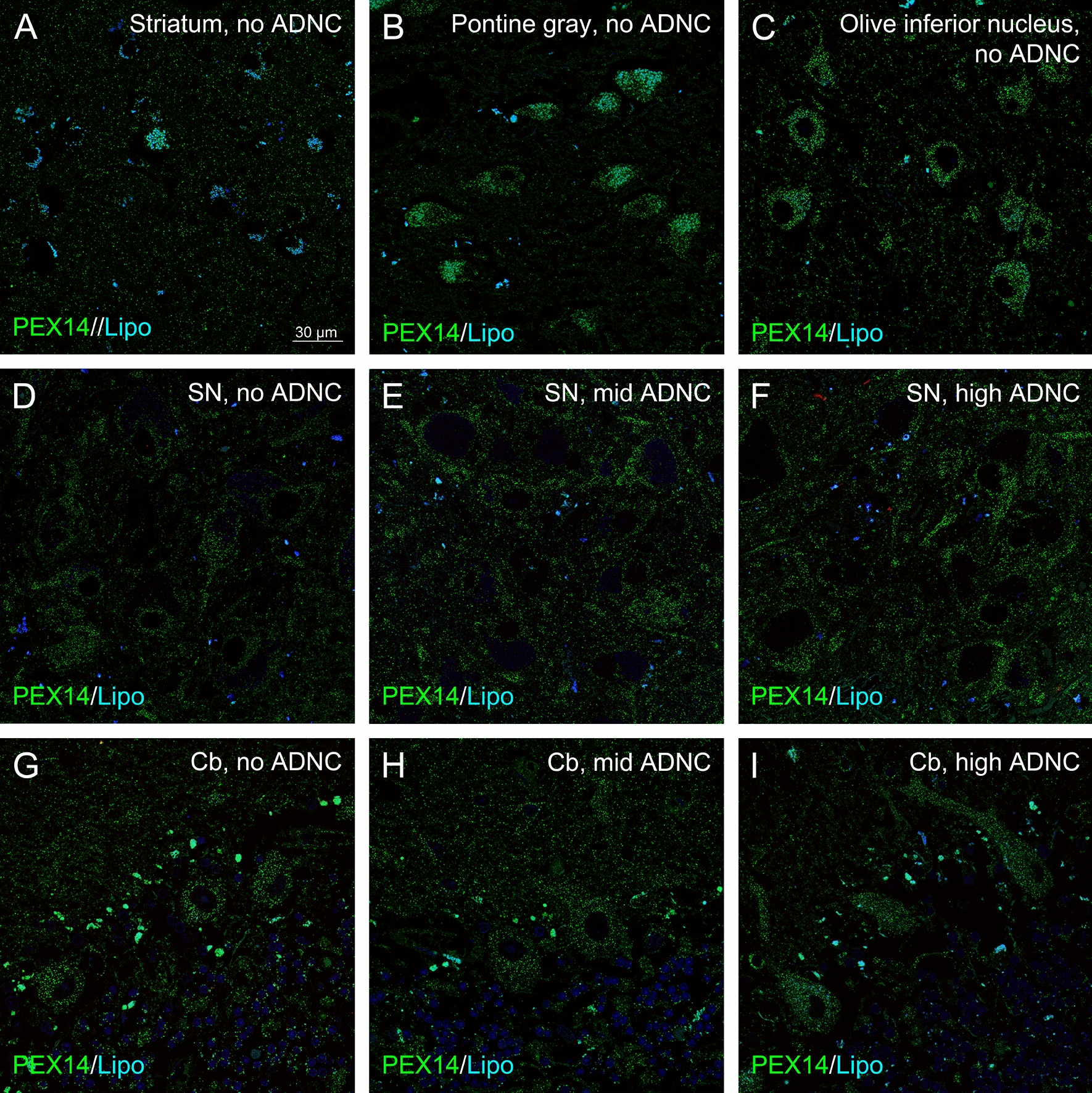
The peroxisome density of neurons varies between different brain areas and during AD-stage progression. Representative photomicrographs of immunostainings for the peroxisomal marker PEX14 (PEX14, green) and autofluorescent lipofuscine granules (turquoise) in neurons of the striatum, pontine gray, inferior olive, substantia nigra (SN) and cerebellum (Cb) of patients with no, mid and high ADNC

**Fig. 9:**
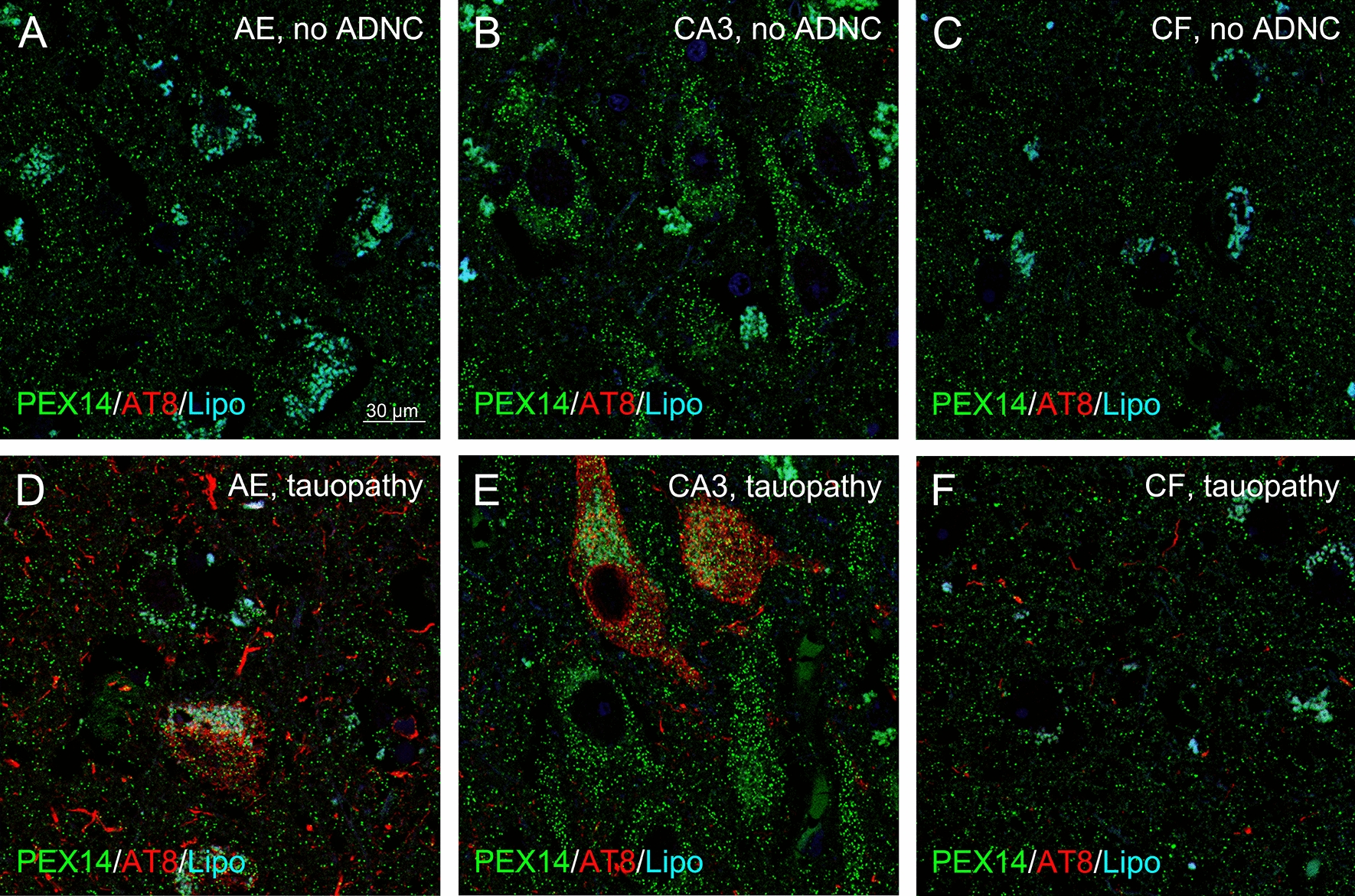
In patients with tauopathy, peroxisome density of pyramidal neurons was higher in the frontal neocortex, but remained unchanged in the hippocampus compared to control patients. Representative photomicrographs of immunostainings for the peroxisomal marker PEX14 (PEX14, green) and hyperphosphorylated tau (AT8, red) together with autofluorescent lipofuscine granules (turquoise) in neurons of area entorhinalis (AE), hippocampal CA3 region (CA3) and the frontal neocortex (CF)

Laser scanning microscopy was performed using the Leica Confocal Laser Scanning Microscope TCS SP2 (Leica, Bensheim, Germany) equipped with a 63 × oil-immersion objective and Leica Confocal Software for image acquisition. Images were taken at the same acquisition settings for the detection of either PEX14 or catalase to ensure comparability of the data between the different patient samples. Fluorescence images stored in the TIF format were imported into Photoshop CS5 for the preparation of representative micrographs (Figs. 2, 4, 6, 8 and 9). In addition, TIF data was imported as an unedited green format into the open-source Java-based image processing program ImageJ for the analysis of the peroxisome density (Figs. 3, 5, and 7). Peroxisome density represents the number of counted particles within the cytosolic area of neurons. Discrimination of peroxisomes from neighboring lipofuscine granules, which all exhibit a strong auto-fluorescence, was achieved using several intermediate steps to isolate peroxisome-sized particles and by setting of the correct particle size (peroxisomes are smaller with 4–50 pixel diameter). Background fluorescence was subtracted by using the automatic “Yen dark” threshold macro function (Additional file 2: Fig. S2). For an automatic analysis of a series of images, an ImageJ macro tool including these pre-processing steps was established (Additional file 2: Fig. S2). Results are presented as box blots overlaid with dot blots, mean values are shown as a black square in the center of the box, median values as a crossbar.

### Statistics

Significant differences between mean values were evaluated either by Wilcoxon test in case of a comparison of two patient groups (e.g. controls vs. tauopathy, Table 2, patients with and without hypercholesterolemia, Additional file 5: Fig. S5) or by one-way analysis of variance (ANOVA-1) and post-hoc Kruskal-Wallis-test in case of a comparison of multiple patient groups (e.g. patient groups with no vs. low vs. mid vs. high ADNC, Table 1; Figs. 3, 5, and 7; Additional file 3: Fig. S3); p-values are given in numbers. For the correlation of the peroxisome density with age or cell size, the correlation coefficient (r) and statistical significance (p) is given in numbers in Additional file 4: Fig. S4. For statistical analysis together with the graphics, we used an R-based self-written program.

**Table 1:** Peroxisome densities of neurons in different brain regions of patients with different ADNC

Region	ADNC	Peroxisome density (number/100 µm^2^)				
		Mean value	Median value	P-value vs no ADNC	P-value vs low ADNC	P-value vs mid ADNC
Striatum	No	14.4	14.3			
	Low	15.6	15.4	n.s		
	Mid	15.7	16.3	n.s	n.s	
	High	13.4	13.6	n.s	0.005	0.004
SN	No	25.9	25.3			
	Low	32.1	33.0	7.6 × 10–6		
	Mid	29.24	31.6	0.024	n.s	
	High	30.1	32.1	0.003	n.s	n.s
Pons	No	20.9	20.6			
	Low	25.3	27.0	2.3 × 10–6		
	Mid	21.0	21.2	n.s	1.7 × 10–4	
	High	21.7	22.0	n.s	4.2 × 10–5	n.s
Inf. Olive	No	13.3	10.6			
	Low	11.7	10.7	n.s		
	Mid	11.8	10.1	n.s	n.s	
	High	12.3	11.4	n.s	n.s	n.s
Cb	No	29.2	29.2			
	Low	28.0	28.0	n.s		
	Mid	26.8	27.0	n.s	n.s	
	High	24.8	24.4	2.4 × 10–4	n.s	0.041

## Results

### Classification of AD pathology according to ABC score

Patients with no positive staining for NFTs (Fig. 1g) and Aβ including neuritic plaques (Fig. 1a) in the frontal neocortex and small amounts of NFTs (Fig. 1f) in the absence of Aβ/neuritic plaques (Fig. 1d) in the hippocampus were considered to have no ADNC (A0/B0-1/C0). In case of medium to high amounts of Aβ/neuritic plaques in the frontal neocortex and hippocampus and missing of NFTs in the frontal neocortex, patients were classified to have low ADNC (A1-2/B1/C1-2). Those with high amounts of Aβ/neuritic plaques (Fig. 1b) combined with low amounts of NFTs (A1-3/B0/C1-3, Fig. 1h) or high amounts of NFTs combined with low amounts of Aβ/neuritic plaques (A1-3/B3/C0-1) in the frontal neocortex (Fig. 1e) as well as medium amounts of Aβ/neuritic plaques and NFTs (Fig. 1K) in the hippocampus were grouped as patients with mid ADNC. When we detected of high amounts of Aβ/neuritic plaques and moderate levels of NFTs in the frontal neocortex (Fig. 1c, l) as well as in the hippocampus (Fig. 1f, l), we classified patients as those with high ADNC (A3/B3/C2-3). In patients with tauopathy, high amounts of NFTs (Fig. 1o), but no Aβ/neuritic plaques (Fig. 1n) can be found in the neocortex and hippocampus (A0/B2/C0) in comparison to high amounts of both Aβ/neuritic plaques (Fig. 1f, m) and NFTs (Fig. 1l) in patients with high ADNC. Details about the abundances of Aβ/neuritic plaques and NFTs in the neocortex and hippocampus for each patient are given in Additional file 6: Table S1.

### Peroxisome density differentially changed in distinct areas of the hippocampus during AD-stage progression

The medial temporal lobe and its sub-region, the transentorhinal cortex, is associated with memory and cognitive function and is one of the early and most severely affected brain areas in AD. Formation of NFTs was initiated in subcortical areas [81] and then spread to the area entorhinalis (pre-clinical stages 1 and 2), subiculum, hippocampus (stages II-IV with mild cognitive impairment) and lastly to neocortical regions (clinical apparent of dementia at stages V-VI) [11]. The opposite is true for the spatiotemporal distribution of Aβ.

Analysis of the peroxisome density of pyramidal neurons (= number of peroxisomes/100 µm^2^ of the cytosolic area) in the area entorhinalis revealed a decrease from patients with no, low, mid and high ADNC (Figs. 2a-c, j and 3a). In the subiculum and CA3 region, we observed an initial increase in patients with low and mid ADNC, but a return to control levels in those with high ADNC (Figs. 2d-i, k-l and 3b, c). Interestingly, the peroxisome density of granule neurons in the dentate gyrus, a region which develop less NFTs in high stages [10], remained nearly constant (Figs. 2m and 3d). Our data suggest an adaptive increase of the peroxisomal compartment failing at later stages of the disease.

In addition, we correlated the peroxisome density with the amount of Aβ plaques or NFTs independent of the ABC score (Additional file 3: Fig. S3). We found a decrease in peroxisome density with increasing levels of Aβ. However, one should bear in mind, that—in the hippocampus—high levels of Aβ are coexisting inevitably with high levels of NFTs. Interestingly, control patients have increasing levels of NFTs with age. As shown in Additional files 4, 6: Fig. S4 and Table S1, the younger patients (number 3, 4 and 6) versus the older patients (number 1 and 8) did not differ with regard to their peroxisome densities. Thus, NFTs seem to be correlated with peroxisome density only in the presence of Aβ plaques. Neuritic plaques were not analysed separately due to their high variability.

### Mild changes in the neocortical peroxisome density during AD-stage progression

Neocortical AD pathology is hallmarked by the formation of extracellular Aβ deposits. They first appeared in one region of the neocortex (most frequently in the frontal and occipital cortices in 83% of all cases followed by the temporal cortex in 66% of all cases and parietal cortex in 33% of all cases; pre-clinical phase), thereafter in all cortices and in the hippocampus, which is only mildly affected (phase 1–2), later in the striatum and diencephalic nuclei (phase 3), brainstem and medulla oblongata (stage 4), and finally in the pontine gray and the molecular layer of the cerebellum (stage 5) [69, 85]). Overall, we measured a small decrease in the peroxisome density of pyramidal neurons in layer III of all cortices (Figs. 4 and 5) in patients with increasing ADNC. Interestingly, neocortical pyramidal neurons have a small size (190 µm^2^) with only half of the peroxisome density (17.3/100 µm^2^) compared those of the hippocampus (ranging from 30–36/100 µm^2^). The size of hippocampal pyramidal neurons varied starting with the smallest ones in the transentorhinal region (170 µm^2^ with a peroxisome density of 31/100 µm^2^) to the ones in the subiculum (242 µm^2^ with a peroxisome density of 30/100 µm^2^) and biggest one in the CA3 region (304 µm^2^ with a peroxisome density of 34/100 µm^2^). Granule neurons in the dentate gyrus have a size of 124 µm^2^ which is even smaller compared to neocortical neurons, but a high peroxisome density of 36/100 µm^2^. The peroxisome density of neocortical pyramidal neurons increased at medium and decreased at high levels of Aβ, but did not change at different levels of NFTs (Additional file 3: Fig. S3). In addition, we analysed, in controls, whether the peroxisome density changes with the cell size (no change, Additional file 4: Fig. S4) or the gender (peroxisome density was 10% and 7% higher in hippocampal and neocortical regions in females compared to males, respectively, Additional file 4: Fig. S4).

### Increased peroxisome density accompanies an increase in catalase only in the frontal neocortex, but not in the hippocampus

Since an increase in peroxisome density does not necessarily mean an increase in its (anti-oxidative) function, we analysed the density of peroxisomes with detectable levels of catalase in a subset of patients (> 80 years of age). In patients with no ADNC, about 25% and 50% of all (PEX14-positive) peroxisomes were catalase-positive in the hippocampus and frontal neocortex, respectively which interestingly also coincides with the hippocampus as starting point in AD. In the hippocampus, either no change (area entorhinalis, Figs. 6a, d, g and 7) or a decrease (subiculum, Fig. 7; CA3 region; Figs. 6b, e, f and 7) in the densities of catalase-positive peroxisomes were found in patients with increasing ADNC. In the frontal neocortex, the density of peroxisomes and of those with high catalase levels increased in parallel during AD-stage progression (Figs. 6c, h, i and 7). Interestingly, in patients above 80 years of age peroxisome density was increased to a higher extent even in the area entorhinalis and frontal neocortex during AD-stage progression (Fig. 7). This suggests an importance of the AD stage for the peroxisomal compartment: old, but not young patients seem to adapt to the disease by increases in the peroxisome density. Indeed, we found a positive correlation of the peroxisome density with age in case of patients with mid and high, but not with no or low ADNC (Additional file 4: Fig. S4).

### During AD-stage progression the peroxisome density increased in pontine gray and the substantia nigra, decreased in the striatum and the cerebellum, and remained unchanged in the inferior olive

Next, we analysed the peroxisome density in brain regions with known high quantities of this organelle [3, 34, 57, 58, 61, 73, 101] which were affected at later stages of AD. Increasing levels of NFTs together with Aβ aggregates have been found at ongoing stages of AD in the striatum, substantia nigra and pontine gray [69]. We observed (i) an initial increase in peroxisome density followed by a return to control levels at later stages in the pontine gray; (ii) an initial increase in peroxisome density which remained constant over time in the substantia nigra; (iii) a decrease in peroxisome density at late stages in the striatum and cerebellum, and (iv) no change in peroxisome density in the inferior olive (Fig. 8; Table 1).

### Peroxisome densities differently changed in different brain areas of patients with tauopathy and decreased in the hippocampus and neocortical regions of patients with hypercholesterolemia

Patients diagnosed for tauopathy contain high amounts of NFTs especially in the entire CA region with first deposits in the parietal-temporal-occipital association cortex in comparison to control patients; all brain regions were free of Aβ (Table 2; Fig. 1n, o). The peroxisome density was higher in tauopathy compared to control patients in the frontal and parietal neocortex; it was lower in the CA3 region, substantia nigra, inferior olive and pontine gray and no differences were found in the striatum, temporal and occipital neocortex, area entorhinalis and the cerebellum (Fig. 9a-d; Table 2).

**Table 2:** Peroxisome densities of neurons in different brain region in patients with tauopathy and controls. Significant differences between the groups were evaluated by Wilcoxon test

Region	Tauopathy	Peroxisome density		
		Mean value	Median value	p-value
AE	No	33.2	31.7	n.s
	Yes	31.9	31.0	
Sub	No	27.6	27.2	n.s
	Yes	29.5	27.8	
CA3	No	35.8	35.3	0.045
	Yes	31.6	32.2	
CF	No	13.0	11.9	
	Yes	17.2	17.6	1.1 × 10–8
CT	No	18.6	18.5	
	Yes	17.0	16.4	n.s
CP	No	17.1	17.0	
	Yes	19.8	20.4	0.003
CO	No	21.9	22.0	
	Yes	22.0	23.8	n.s
Striatum	No	14.0	13.3	
	Yes	15.2	15.0	n.s
SN	No	28.7	29.6	
	Yes	21.3	26.7	4.9 × 10–6
Pons	No	23.2	24.3	
	Yes	16.9	16.0	5.6 × 10–7
Inf. Olive	No	15.3	12.1	
	Yes	9.9	9.4	2.8 × 10–5
Cb	No	29.1	29.1	
	Yes	29.3	29.2	n.s

AD is often associated and probably accelerated with co-morbidities such as hypertension, diabetes mellitus type II, hypercholesterolemia, and cerebral angiopathy. To exclude that co-morbidities and not the presence of Aβ plaques and NFTs or the combination of both caused changes in peroxisome density during AD-stage progression, we analysed peroxisome density in relation to these diseases independent of the ABC score. As nearly all patients (except of two patients) are hypertensive (Additional file 7: Table S2), no evaluation of the influence of a high blood pressure on peroxisome density was possible. With regard to diabetes mellitus type II which is frequently accompanied by angiopathy and thus, a reduced blood (nutrient) supply and damage of neurons, we observed an increase in the peroxisome density in the hippocampus and neocortical regions (data not shown)—probably an adaptive response. Instead, in patients with hypercholesterolemia, the peroxisome density—similar to patients with high ADNC—was lower when compared to control patients (Additional file 5: Fig. S5). Interestingly, patients with cerebral angiopathy (diagnosed for micro- and macroangiopathy) and those with amyloid angiopathy show almost identical peroxisome densities in the frontal neocortex (data not shown). Interestingly, amyloid angiopathy is frequently linked to AD [38].

## Discussion

Postmortem analysis of human brain samples revealed a differential temporal as well as spatial response of the peroxisomal compartment during AD-stage progression as defined by neuropathological changes. The characteristic differences were (i) a continuous decrease in the peroxisome density in the area entorhinalis, (ii) an initial increase and a decrease at late stages of the disease in the hippocampus and (iii) an increase at early stages of AD in the frontal neocortex and exclusively in this area it was accompanied by an increase in catalase. We assume that the observed changes in peroxisome density could represent an adaptive neuroprotective or pathogenic response induced by the differential appearance of Aβ plaques and NFTs. In the following, we would like to discuss changes in organelle abundances during aging (i) and in neurodegenerative diseases (ii), the relationship between peroxisomal density, oxidative stress and inflammation (iii), region-specific differences in the response of peroxisomes (iv), and the relationship between peroxisome density and other co-morbidity factors (v) during AD-stage progression.

### Possible mechanisms for changes in the peroxisomal density during aging

The homeostasis of the number of organelles in different cell types is regulated by their biogenesis, proliferation and degradation (e.g. autophagocytosis). Alterations in numerical abundance are induced by diverse stresses which is well-known for mitochondria that either proliferated due to nutritional adaptation [20] or are fragmented and eliminated by mitophagy after toxic damage [99]. Similar to mitochondria, the peroxisomal compartment can rapidly adapt to changing cellular conditions such as increased levels of endogenous [65] and nutritional lipids, growth factors, cytokines [37, 91] or when treated with hypolipidemic drugs [39]. In this case altering of the number of organelles and their metabolic function is mediated through activation of the nuclear receptor peroxisome proliferator-activated receptor α (PPARα) and PEX11α gene expression [7, 23, 75], whereas the basal number of peroxisomes is regulated by the PEX11β gene [2, 50].

Alterations in peroxisome density are supposed to occur during aging. Previous studies showed a lowering of catalase and of the β-oxidation enzyme acyl-CoA oxidase 1 together with an increase in thiolase A and urate oxidase protein levels in 39-months compared to 2-months old rats [7, 64, 100]. In humans, lipid profile analysis revealed a stable lipidome during normal brain ageing with a minor decrease in level of polyunsaturated fatty acids (PUFAs) solely in the entorhinal cortex indicating a region-specific reduction of the peroxisomal β-oxidation [60]. A decrease in catalase was found in senescent versus young fibroblasts [43] which may lead to oxidative stress accompanied by peroxisome proliferation as shown by an increased number of PEX14-positive peroxisomes [49, 83]. Consistently, exogenous addition of hydrogen peroxide to HepG2 cells induced tubulation of peroxisomes which is suggested as a pre-form for peroxisome proliferation via PEX11β [76]. Moreover, with age, PTS1-mediated import efficiency is impaired via oxidative damage which leads to lower levels of catalase in comparison to less affected peroxisomal lipid metabolism enzymes resulting in an imbalance towards pro-oxidant reactions [83]. In fact, in hypocatalasemic human fibroblasts with approximately 25% residual peroxisomal catalase content, long-term hydrogen peroxide production accelerated the development of age-related diseases [97].

### Possible mechanisms for changes in the peroxisomal density in neurodegenerative diseases

The peroxisomal numerical abundance and metabolic function was also supposed to play an important role in neurodegenerative diseases [17]. Considering the regional spread of AD pathologies, we observed a constant decrease in peroxisome density during AD in the early affected area entorhinalis and an initial increase returning to control levels in the later affected subiculum, CA3 region and frontal neocortex. The gradual decrease in peroxisome density during AD-stage progression in the area entorhinalis might be the consequence of a reduced peroxisomal lipid metabolism and long-term oxidative stress. This idea is supported by data of Kou et al. [48] showing elevated levels of VLCFAS in the transentorhinal cortex and decreased levels of plasmalogens in areas with high amounts of NFTs. Similarly, brain DHA levels were most prominently reduced in the hippocampus of AD patients [45]. Interestingly, inhibition of peroxisomal β-oxidation of VLCFAs increased the synthesis of Aβ in the rat brain [78]. Aβ and NFTs inhibited ER-associated degradation of misfolded proteins [13] thereby activating peroxisomal Lon protease LonP2 and pexophagy [66]. Long-term oxidative stress together with a reduced nutrient supply due to extracellular Aβ deposits [51] induced autolysis as well as NBR1-dependent pexophagy [27]. Moreover, NFTs and Aβ aggregates inhibit PINK1 and Parkin, both positive regulators of mitophagy [26] which coincided with a loss of peroxisomes [82]. Thus, the combination of high levels of VLCFAs, oxidative stress and Aβ and NFTs at late stages of AD might induce the degradation and thus lower density of peroxisomes.

Our findings in the hippocampus and frontal cortex, where we detected an initial increase in the peroxisome density and a return to control levels at late AD stages confirmed two animal studies. In the hippocampus of transgenic AD mice, peroxisome abundance (by measuring the PEX14 protein level) increased in the first 3 months and returned to control levels at 6 months of age. The levels of ABCD3/PMP70, catalase and SOD2 proteins changed in parallel to PEX14, and those of SOD1 and GPX decreased with time [24]. Similarly, when cortical neurons were treated with Aβ the number of peroxisomes (by measuring the ABCD3/PMP70 protein level) increased after 6 days followed by a decrease at 14 days in vitro. Acyl-CoA oxidase 1, superoxide dismutase (SOD)1 and PPARα changed accordingly, but those of catalase, SOD2 and thiolase A constantly decreased together with increased ROS production [16]. These additional data again suggests mild oxidative stress and a reduced peroxisomal metabolism in the beginning of AD leading to high levels of VLCFAs and low levels of DHA and hydroxy-DHA and all these factors are known to stimulate peroxisome proliferation [52]. In the human brain, only Kou et al. [48] analysed changes in peroxisome density during AD-stage progression. They analysed the soma and processes of neurons in the frontal cortex and observed an increased peroxisome density in the soma, but only in areas with NFTs. In contrast, in our study, the peroxisome density in the frontal cortex was the same in control and AD patients. The use of different organelle markers—ABCD3/PMP70 by Kou et al. [48] and PEX14 in our study—might be the reason for the different findings. Since PMP70 is involved in shuttling of lipids into the peroxisomal matrix for degradation, the increase in the density of ABCD3/PMP70-positive peroxisomes might be a compensatory mechanism to the increased level of VLCFAs found in the frontal cortex of AD patients [48]. Thus, ABCD3/PMP70, in contrast to PEX14, does not label all peroxisomes due to metabolic and maturation heterogeneities of individual organelles and may increase although the overall peroxisome abundance remains unchanged. In addition, we evaluated the number and not the area of organelles per area—an increased amount of ABCD3/PMP70 of individual peroxisomes lead to stronger and broader fluorescence signal mimicking an increase of the area, while the number remains constant.

Furthermore, dysfunction of peroxisomes in glial cells may affect neuronal function and thus be involved in AD. A dysfunction of peroxisomal function in astrocytes increased the level of VLCFAs in myelin, but this does not affect neuronal function [9]. Since peroxisomes are rare in the neuronal axon [5], those of the oligodendrocytes, are thought to sustain axonal integrity and function [5, 46], e.g. by providing plasmalogens and lipids for rapid impulse conduction [5]. Consistently, loss of peroxisomal function in oligodendrocytes leads to axon degeneration, demyelination and neuroinflammation [46]. In microglia cells, reversing the decrease of peroxisomal proteins such as PMP70, PEX11β, PEX5 and catalase during inflammatory reactions, improved molecular, morphological and behavioral outcome in mice [68].

Interestingly, the peroxisome and its abundance has just started to be considered as a possible target for the therapy of neurodegenerative diseases including Parkinson disease, Alzheimer´s disease, Huntington disease, amyotrophic lateral sclerosis and multiple sclerosis [1]. The peroxisome proliferator phenylbutyrate reversed the inflammation-induced decrease in ABCD3/PMP70, PEX11β and catalase protein levels and is recommended as a possible drug for treatment of multiple sclerosis [68]. In addition, PPARα and PPARγ agonists showed anti-inflammatory and energy balancing effects in AD patients [1] and thus may slow down AD-stage progression [71, 89, 96]. Indeed, especially high levels of PPARγ reduced Aβ deposition and the production of pro-inflammatory cytokines [94].

### Possible relationship between peroxisomal metabolism, oxidative stress, and inflammation in AD-stage progression

Oxidative stress has been suggested to be one of the main causes of AD pathology [13]. For example, Aβ oligomers contain entrapped metal ions and thus produce ROS [14] and the induction of ROS-sensitive pathways promote formation of NFTs. Aβ and tau promote the aggregation of each other which causes inhibition of macroautophagy ending up in a vicious cycle accelerating the progression of the disease. A dysfunction of peroxisomes is possibly the source of oxidative stress in the beginning of AD, for example, a reduced synthesis of plasmalogens and PUFAs—both necessary for trapping ROS-increase the levels of oxidized lipids in cellular membranes. Moreover, the downregulation of ABCD3/PMP70 [48] could interfere with the transport of oxidized lipid derivatives into peroxisomes, thus hindering their degradation via β-oxidation.

In addition, glial and microglial cells release pro-inflammatory cytokines (e.g. IL-1, IL-6, TNFα) [98] and these factors upregulate Aβ production and hyperphosphorylation of tau thereby amplifying AD-stage progression [22]. Disturbances in the peroxisomal function could be again a causative factor as peroxisomes are responsible for the degradation of pro-inflammatory lipid derivatives, e.g. prostaglandins [74] and leukotrienes [43, 54]. As a secondary vicious cycle, increased levels of TNFα decrease catalase protein level [68] which in turn lowers peroxisomal β-oxidation [36, 91] and thus increase the levels of oxidized lipids and pro-inflammatory factors.

### Region-specific differences in the response of peroxisomes during AD-stage progression

Interestingly, only the neocortex, but not the hippocampus adapted to AD-induced oxidative stress by an increase in the density of catalase-positive peroxisomes. In addition to catalase, such brain region-specific differences have been described also for the levels of other antioxidant enzymes in control and AD brains. Consistent with our results, catalase [15], and PRDX5 [33] levels were high in the frontal neocortex, pons and cerebellum in comparison to low levels in the hippocampus and substantia nigra, whereas similar levels in all brain regions were measured for SOD1, SOD2, peroxiredoxin (PRDX)6 and glutathione peroxidase [15, 67]. Consistent with our findings, the level of catalase increased during AD in the neocortex and only to a lesser extent in the hippocampus [15]. The same was true for neuronal PRDX2 [72]. No compensatory response to AD was found for the antioxidant enzymes SOD1, SOD2, glutathione peroxidase [15], PRDX1, PRDX3, PRDX4 and PRDX6 [47]. In addition, the lower antioxidant defense of the hippocampus would fit to the early atrophy of this brain region in AD [77]. Region-specific differences in AD do not only exist for the antioxidant defense systems, but also for tau and Aβ, which are both transformed from monomeric forms into aggregates. Whereas aggregation of Aβ begins in the neocortex, hyperphosphorylation and aggregation of tau starts in the hippocampus and occurs later in the neocortex. Interestingly, tau stabilizes axonal transport of organelles and other cell components [41] which is inhibited upon tau hyperphosphorylation occurring in AD [59]. As a consequence, organelles—including peroxisomes—accumulate in the soma and proximal dendrites [80] and they are less abundant in more distal neuronal processes for the detoxification of ROS and for providing precursors of cholesterol and membrane lipids, for membrane formation of synpases and vesicles [12] leading to a decline in neuronal function [35].

### Possible relationship between peroxisomal metabolism, AD-stage progression and co-morbidities

Next, we analysed whether co-morbidity factors, in addition to Aβ and NFTs, might affect peroxisome densities. Whereas diabetes mellitus type II had no influence on AD-induced changes in peroxisome abundance, we found a decrease thereof in the hippocampus and frontal neocortex in patients with hypercholesterolemia at mid and late stages of AD (Additional file 5: Fig. S5). Formerly, it was assumed that there is no link between AD and plasma cholesterol levels, since very different blood cholesterol levels were found in patients with AD and cholesterol metabolism in brain and in peripheral tissues is independent from each other [32]. However, a retrospective 3-year multicenter study revealed that higher levels of LDL cholesterol in the blood are certainly associated with a higher percentage of early onset AD pathology [95]. Interestingly, AD patients contain higher plasma levels of oxysterols (mainly due to auto-oxidation of cholesterol), e.g. 7β-hydroxycholesterol, 7-ketocholesterol, 27S-hydroxycholesterol and 24S-hydroxycholesterol [84], which all can pass the blood-brain barrier, enter the brain parenchyma and there may lead to oxidative stress and especially 7β-hydroxycholesterol has been shown to reduce peroxisome number in murine C2C12 myoblasts [29]. Vice-versa, AD can cause an increase in plasma cholesterol. In the brain of AD patients, the activity of cholesterol 24-hydroxylase (Cyp46A1) is increased [8] indicating excess amounts of cholesterol in neurons which is thought to aggravate AD pathology [25, 90]. 24S-hydroxycholesterol can diffuse to glial cells, but mainly crosses the blood-brain barrier. In the brain as well as in the liver it is metabolized into bile acids to be secreted with feces [70]. However, 24S-hydroxycholesterol activates the nuclear receptor LXR, which upregulates IDOL gene expression which leads to a degradation of the LDL receptor in the liver [102] and increases the plasma cholesterol level [40].

**Fig. 10:**
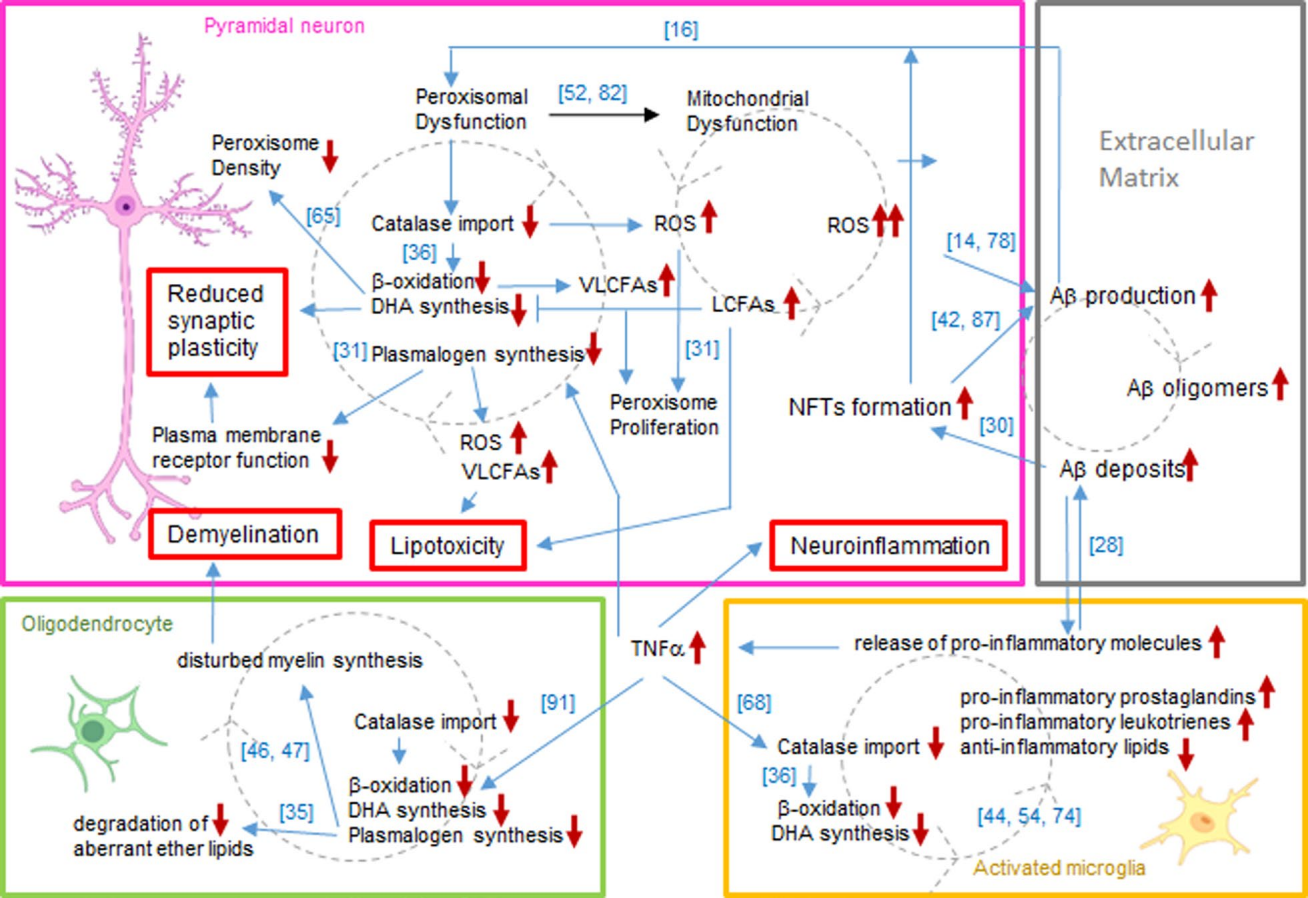
Vicious cycles in AD pathogenesis involving Aβ and NFT formation as well as disturbances in peroxisomal and mitochondrial metabolic pathways. In neurons (pink box), peroxisomal dysfunction includes reductions in catalase import, peroxisomal β-oxidation, and DHA and plasmalogen synthesis. This caused oxidative stress, accumulation of LCFAs/VLCFAs (exerting lipotoxicity) and changes in plasma membrane fluidity (exerting reduced synaptic plasticity) accompanied by an initial increase and a later fall in peroxisome numerical abundance. The resulting oxidative stress in turn elevates Aβ production, its aggregation and the formation of NFTs promoting the further disturbance in peroxisomal and mitochondrial dysfunction. Microglial cells (yellow box) are activated by Aβ and release TNFα, which also impairs catalase import, β-oxidation and DHA synthesis resulting in an imbalance of pro- and anti-inflammatory molecules thereby maintaining brain inflammation. TNFα-induced disturbance of peroxisomal function in oligodendrocytes (green box) affects myelin composition finally leading to demyelination, axon loss and a reduced synaptic transmission. The described vicious cycles are depicted in gray dotted lines with arrows indicating the rotation direction. Bold red arrows exemplify alterations (increases ↑ and decreases ↓); blue arrows represent the sequences of different processes with indications of reference numbers of corresponding publications in square brackets. Figure is created with BioRender.com

In conclusion, our data suggest that distinct factors such as oxidative stress and disturbances in lipid and cholesterol metabolism account for the different changes in peroxisome density as well as its heterogeneity in different areas of the brain, e.g. hippocampus versus neocortex, during AD-stage progression (Fig. 10). We assume that the changes in peroxisome density reflect an initial stress response at early stages—it remains open and has to be clarified by future studies whether this is neuroprotective or pathogenic—and a decompensation thereof at later stages of AD. Further investigations on these aspects might help to develop new strategies to inhibit the decline of peroxisomes to slow down the progression of AD and other neurodegenerative diseases.

## Supplementary Information

**Additional file 1**: **Fig. S1**. Excision of the respective brain areas followed a defined pattern as shown for the neocortical areas, the striatum, substantia nigra and the cerebellum. Within the hippocampal section, we analyzed the area entorhinalis, subiculum and CA3 region. Colored images were taken from the Interactive Atlas viewer.

**Additional file 2**: **Fig. S2**. Description of the steps of the ImageJ macro tool to quantify peroxisomesand the cytosolic areain neuronal cell bodies.

**Additional file 3**: **Fig. S3**. The peroxisome density of pyramidal neurons decreased in relation to the amount of Aβ, but not of NFTs. Data were obtained using PEX14 immunofluorescence images running through a self-written ImageJ macro-tool for counting particles and measuring the cytosolic area. Ten individual neurons were analysed from each patient and plotted as a point on the graph. Crossbar = median value, black diamond in the box center = mean value, vertical lines above and below each box = SD values, only statistically significant differences between distinct groups were shown and labelled with the respective p-values.

**Additional file 4**: **Fig. S4**. The peroxisome density of hippocampal and neocortical pyramidal neurons increased as a function of age in AD patients, but not in healthy controls. Data were obtained using PEX14 immunofluorescence images running through a self-written ImageJ macro-tool for counting particles and measuring the cytosolic area. Data of patients with no, low, midand highADNC of hippocampaland neocorticalpyramidal neurons were related to the age and cell size. The correlation coefficient for each patient group is given at the left site in the respective color.

**Additional file 5**: **Fig. S5**. A lower peroxisome density of hippocampal und neocortical pyramidal neurons was detected in patients with in comparison to those without hypercholesterolemia especially at mid and late stages of AD. Data were obtained using PEX14 immunofluorescence images running through a self-written ImageJ macro-tool for counting particles and measuring the cytosolic area. Ten individual neurons were analysed from each patient and plotted as a point on the graph. Crossbar = median value, black diamond in the box center = mean value, vertical lines above and below each box = SD values.

**Additional file 6**: **Table S1**. Patient data including gender, age, ABC score and amount of Aβ and NFTs in the frontal, parietalor occipitalneocortex, area entorhinalis, subiculumand CA3 region of the hippocampal formation. A = Aβ plaques, A0 = no, A1 = low amount in the CF and AE, A2 = intermediate amount in CF, AE and Sub, A3 = high amount in CF, AE, Sub and all CA regions; B = NFTs, 0 = no, B1 = low amount in the transentorhinal cortex and AE, B2 = intermediate amount of NFTs in the hippocampal formation, B3 = high amount of NFTs in the hippocampal formation; C = neuritic plaques, C0 = no, C1 = sparse, C2 = moderate, C3 = frequent.

**Additional file 7**: **Table S2**. Cause of death, clinical data, and co-morbidities of the patients. For each disease or symptom, the number of the patientsis listed according to the ADNC.

## Data Availability

All data generated or analysed during this study are included in this published article and its supplementary information files.

## Abbreviations

Aβ: Amyloid-β
AE: Area entorhinalis
AD: Alzheimer´s disease
ADNC: Alzheimer´s disease neuropathological changes
BSA: Bovine serum albumin
CA3: CA3 band of the hippocampus
Cb: Cerebellum
CF: Frontal neocortex
CP: Parietal neocortex
CO: Occipital neocortex
CT: Temporal neocortex
DHA: Docosahexanoic acid
FFPE: Formalin-fixed paraffin-embedded
NFTs: Neurofibrillary tangles
PBS: Phosphate-buffered saline
PMPs: Peroxisomal membrane proteins
PPARs: Peroxisome proliferator-activated receptors
PRDX: Peroxiredoxin
PUFAs: Polyunsaturated fatty acids
ROS: Reactive oxygen species
SN: Substantia nigra
SOD: Superoxide dismutase
Sub: Subiculum
VLCFAs: Very-long chain fatty acids
