# Supplementary material for: Rise and fall of peroxisomes during Alzheimer´s disease: a pilot study in human brains
Source: Acta Neuropathol Commun. 2023 May 11;11:80. doi: 10.1186/s40478-023-01567-0 (PMC10176950; doi:10.1186/s40478-023-01567-0)
Supplement: Supplementary file 6 — Additional file 6: Table S1. Patient data including gender, age, ABC score and amount of Aβ and NFTs in the frontal, parietalor occipitalneocortex, area entorhinalis, subiculumand CA3 region of the hippocampal formation. A = Aβ plaques, A0 = no, A1 = low amount in the CF and AE, A2 = intermediate amount in CF, AE and Sub, A3 = high amount in CF, AE, Sub and all CA regions; B = NFTs, 0 = no, B1 = low amount in the transentorhinal cortex and AE, B2 = intermediate amount of NFTs in the hippocampal formation, B3 = high amount of NFTs in the hippocampal formation; C = neuritic plaques, C0 = no, C1 = sparse, C2 = moderate, C3 = frequent. [file 40478_2023_1567_MOESM6_ESM.docx]

|  |  |  | |  | |  | |  | |  | |  | |  | |  | |  | |  |
| --- | --- | --- | --- | --- | --- | --- | --- | --- | --- | --- | --- | --- | --- | --- | --- | --- | --- | --- | --- | --- |
| Patient | Sex/Age | ABC score | | CF Aβ | | CF NFTs | | AE Aβ | | AE NFTs | | Sub Aβ | | Sub NFTs | | CA3 Aβ | | CA3 NFTs | |  |
| No ADNC | | |  | |  | |  | |  | |  | |  | |  | |  | |  | |
| 1 | f/83 | A0B1 | | (-) | | (-) | | (-) | | (++) | | (-) | | (++) | | (-) | | (+) | |  |
| 2 | f/73 | A0B1 | | (-) | | (-) | | (-) | | (+) | | (-) | | (+) | | (-) | | (+) | |  |
| 3 | f/67 | A0B0 | | (-) | | (-) | | (-) | | (+) | | (-) | | (-) | | (-) | | (-) | |  |
| 4 | f/64 | A0B0 | | (-) | | (-) | | (-) | | (+) | | (-) | | (-) | | (-) | | (-) | |  |
| 5 | f/71 | A0B1 | | *(-) | | *(-) | | (-) | | (++) | | (-) | | (++) | | (-) | | (++) | |  |
| 6 | m/67 | A0B0 | | (-) | | (-) | | (-) | | (-) | | (-) | | (-) | | (-) | | (-) | |  |
| 7 | m/76 | A0B1 | | (-) | | (-) | | (-) | | (++) | | (-) | | (++) | | (-) | | (-) | |  |
| 8 | m/84 | A0B1 | | (-) | | (-) | | (-) | | (+) | | (-) | | (++) | | (-) | | (++) | |  |
| 9 | m/75 | A0B0 | | (-) | | (-) | | (-) | | (-) | | (-) | | (-) | | (-) | | (-) | |  |
| 10 | m/77 | A0B1 | | (-) | | (-) | | (-) | | (+) | | (-) | | (++) | | (-) | | (++) | |  |
| Low ADNC | | |  | |  | |  | |  | |  | |  | |  | |  | |  | |
| 11 | f/60 | A1B1 | | (+) | | (-) | | (++) | | (++) | | (+) | | (+) | | (-) | | (-) | |  |
| 12 | f/79 | A1B1 | | (+++) | | (-) | | (-) | | (+++) | | (-) | | (+++) | | (-) | | (+) | |  |
| 13 | f/78 | A1B0 | | (++) | | (-) | | (+) | | (-) | | (-) | | (-) | | (-) | | (-) | |  |
| 14 | f/86 | A1B1 | | (++) | | (-) | | (+) | | (+) | | (+) | | (++) | | (-) | | (-) | |  |
| 15 | m/68 | A2B1 | | **(++) | | **(-) | | (+) | | (-) | | (+) | | (-) | | (-) | | (++) | |  |
| 16 | m/84 | A1B1 | | (++) | | (-) | | (-) | | (++) | | (-) | | (++) | | (-) | | (++) | |  |
| 17 | m/73 | A2B1 | | (+++) | | (-) | | (++) | | (+) | | (++) | | (+) | | (+) | | (-) | |  |
| 18 | m/70 | A2B1 | | (++) | | (-) | | (++) | | (+) | | (++) | | (+) | | (-) | | (+) | |  |
| Mid ADNC |  |  | |  | |  | |  | |  | |  | |  | |  | |  | |  |
| 19 | f/74 | A1B2 | | (+++) | | (+) | | (++) | | (++) | | (+) | | (+) | | (+++) | | (+++) | |  |
| 20 | f/94 | A3B2 | | (+++) | | (+) | | (+++) | | (+++) | | (++) | | (+++) | | (+) | | (++) | |  |
| 21 | f/87 | A3B2 | | (+++) | | (+) | | (+++) | | (++) | | (++) | | (++) | | (+) | | (++) | |  |
| 22 | m/84 | A2B2 | | (+++) | | (+) | | (+) | | (+) | | (+) | | (+) | | (-) | | (-) | |  |
| 23 | m/84 | A1B2 | | (+++) | | (++) | | (-) | | (++) | | (+++) | | (-) | | (-) | | (+++) | |  |
| 24 | m/72 | A3B2 | | (+++) | | (+) | | (+++) | | (++) | | (+++) | | (++) | | (-) | | (+) | |  |
| 25 | m/87 | A1B2 | | (+++) | | (+) | | (+) | | (+++) | | (+) | | (+++) | | (-) | | (+++) | |  |
| High ADNC |  |  | |  | |  | |  | |  | |  | |  | |  | |  | |  |
| 26 | f/64 | A3B3 | | (+++) | | (+++) | | (+++) | | (+++) | | (+++) | | (+++) | | (+++) | | (+++) | |  |
| 27 | f/82 | A3B3 | | (+++) | | (++) | | (++) | | (+++) | | (+++) | | (+++) | | (++) | | (+++) | |  |
| 28 | f/75 | A3B3 | | (+++) | | (+++) | | (++) | | (+++) | | (+++) | | (+++) | | (++) | | (++) | |  |
| 29 | m/71 | A3B3 | | (+++) | | (+++) | | (+++) | | (+++) | | (+++) | | (+++) | | (++) | | (++) | |  |
| 30 | m/89 | A2B3 | | (++) | | (++) | | (+) | | (+++) | | (++) | | (+++) | | (++) | | (+++) | |  |
| 31 | m/80 | A3B3 | | (+++) | | (+++) | | (++) | | (+++) | | (++) | | (+++) | | (++) | | (+++) | |  |
| 32 | m/80 | A3B3 | | (+++) | | (+++) | | (+++) | | (++) | | (+++) | | (+++) | | (++) | | (++) | |  |
| Tauopathy |  |  | |  | |  | |  | |  | |  | |  | |  | |  | |  |
| 33 | f/84 | A0B2 | | (-) | | (+) | | (-) | | (+) | | (-) | | (++) | | (-) | | (++) | |  |
| 34 | f/74 | A0B2 | | (-) | | (-) | | (-) | | (++) | | (-) | | (+) | | (-) | | (++) | |  |
| 35 | f/75 | A0B2 | | (-) | | (+) | | (-) | | (+++) | | (-) | | (+++) | | (-) | | (+++) | |  |
| 36 | m/75 | A0B2 | | (-) | | (+) | | (-) | | (+) | | (-) | | (++) | | (-) | | (++) | |  |
| 37 | m/78 | A0B2 | | (-) | | (-) | | (-) | | (+) | | (-) | | (++) | | (-) | | (++) | |  |
| 38 | m/82 | A0B2 | | (-) | | (-) | | (-) | | (++) | | (-) | | (++) | | (-) | | (+++) | |  |
| *CO | **CP |  | |  | |  | |  | |  | |  | |  | |  | |  | |  |
