# Supplementary material for: Rise and fall of peroxisomes during Alzheimer´s disease: a pilot study in human brains
Source: Acta Neuropathol Commun. 2023 May 11;11:80. doi: 10.1186/s40478-023-01567-0 (PMC10176950; doi:10.1186/s40478-023-01567-0)
Supplement: Supplementary file 7 — Additional file 7: Table S2. Cause of death, clinical data, and co-morbidities of the patients. For each disease or symptom, the number of the patientsis listed according to the ADNC. [file 40478_2023_1567_MOESM7_ESM.docx]

|  | Patients with |  |  |  |  |
| --- | --- | --- | --- | --- | --- |
| **Cause of death** | no ADNC (1-10) | low ADNC (11-18) | mid ADNC (19-25) | high ADNC (26-32) | Tauopathy (33-38) |
| Cardiovascular diseases (lung embolism, heart failure) | 1, 2, 3, 4, 5, 7 | 11, 13, 15, 16 | 19, 21, 22, 23 |  | 34, 35 |
| Cardiogenic shock |  |  |  | 30 | 36 |
| Chronic pulmonary disease | 6, 10 | 14, 18 |  |  | 38 |
| Ischemic stroke | 7 |  | 20 | 32 |  |
| Multiple organ failure | 9 | 12, 18 | 19 | 28, 31 | 37 |
| Cancer | 4, 6, 8 | 16, 17 | 24 | 27, 28, 29 | 33 |
| Perioperative death | 2 |  | 25 |  | 35 |
| Unknown |  |  |  | 26 |  |
|  |  |  |  |  |  |
| **Clinical findings - brain** |  |  |  |  |  |
| Intracranial hemorrhage | 3 |  | 20 | 26, 29 |  |
| Ischemic stroke | 7 | 12, 14, 17 | 19, 21, 22 | 32 |  |
| Tumor | 1 |  |  |  |  |
| Amyloidangiopathy | 6 |  |  |  |  |
| Atrophy, neurodegeneration | 5, 8 | 13, 16, 17, 18 | 24, 25 | 26, 27, 28, 30 | 34, 37, 38 |
| Increased intracranial pressure | | 11, 15 | 23 |  | 33, 35, 36 |
|  |  |  |  |  |  |
| **Diseases** |  |  |  |  |  |
| Heart failure |  | 13, 14, 15 | 19, 21, 22 |  | 35, 36, 38 |
| Heart valve disease | 2 |  |  | 32 | 36 |
| Pulmonary hypertension | 10 | 11 | 22 |  |  |
| Chronic obstructive pulmonary disease, | 4, 5, 10 | 15 | 19 |  |  |
| Diabetes-related renal failure | 9 | 12 |  |  | 38 |
| Rheumatoid arthritis |  |  | 19 |  |  |
| Cancer | 4, 6, 7, 8 | 17, 18 | 24 | 27, 28 | 37 |
